# Should the Use of White Lead as a Paint Be Forbidden by Public Authority?

**Published:** 1853-03

**Authors:** 


*Should the use of White Lead as a Paint be forbidden by Public Authority?*—This question is exciting considerable interest in France, one of the few countries in Europe where a due regard to the public health is part of the business of government. In England, and this country, we are too jealous of individual rights, too independent, if you please, to allow our rulers to watch over the well-being of the community.

We shall therefore merely present facts, without comment, as given to us in a memoir of Dr. H. De Castelnau:

"In his remarkable memoir on *Painting with White Zinc*, Dr. Bouchon advised the government, if it had due regard for the health of workmen, to forbid the use of white lead as a paint on all the public buildings, and that an example should be presented for imitation by the substitution of an article less deleterious. The favorable manner in which this proposition was received by the Academy of Medicine, at its session on the 4th of November, 1837, indicates their full accordance in the idea, although they were necessarily restrained from entering into the merits of the question of economics, and we derive a similar indication of opinion in the large premium bestowed by the Institute, in 1849, on M. Le Claire, for his essay on the means of rendering occupations less unhealthy.

All these circumstances have doubtless tended to aid in diffusing a report that government is about suppressing the manufacture of white lead. To aid such a measure, a few details on the point of sickness and mortality will be of use.

In accordance with a requisition from the prefect of Police, the administration of hospitals demanded an annual return of all cases admitted into

---

them of diseases from lead. It thus appears, that during ten years, (1838 to 1847,) 3142 were admitted, and that 112 of these died, being an annual mean of 314 sick and 11 dying. There can, however, be scarcely a doubt that the first number is too low. There is very frequently a doubt as to the nature of the complaint on admission—indeed, lead affections take some time to develop themselves, and thus cases are frequently referred to other diseases. It is highly probable that at least 400 cases are annually admitted, and that fifteen deaths occur.

Of the gross number, (3142,) three-fifths (1898) were cases of workmen engaged in the manufacture of white or red lead, and the remaining two-fifths were persons employed in using these products, as painters, grinders, makers of porcelain cards, (so called,) &c.

Then again, there are many cases treated at their own dwellings, but unfortunately we have no data exactly to estimate their number. It is quite probable that they are at least equal to those treated in hospitals, and if this be conceded, we have annually 400 cases of lead disease in those who are strictly manufacturers of the preparations of lead, and of which 14 die. It would be too extravagant to carry this proportion throughout France. Reducing it nine-tenths, and with a due regard to the statistics of provincial hospitals, we are certainly safe in stating the total annual result at 2000 cases of disease and 80 deaths. These would be at an end with the suppression of the manufacture.

But there is another matter to be also considered. The average sojourn of a patient with saturnine disease in a hospital is sixteen days. Add to this the illness and loss of time that precedes, and the debility, broken health, and loss of business that follows so many of the workmen. Even if we do not estimate this last, still, the hospitals will be relieved annually of sixteen or seventeen thousand days of sick persons, not to take into account the permanent residence of many incurables.

Can there, then, be a doubt that the public health will be greatly improved by the suppression of these manufactories? Still, however powerful may be the arguments in favor, it would hardly answer to attempt their suppression, unless we could find a proper substitute, both in the healthiness of its manufacture, and its value in the arts. Can both of these objects be accomplished by the employment of the white oxide of zinc? *(le blanc de zinc?)*

As to the first, Dr. Bouchut, just at the time of concluding his memoir, in July, 1851, received the following return from the company manufacturing zinc at Asnieres. Up to the date named, they had employed 151 workmen, who together had performed labor during 31,585 days, and had been in the factory 36,156 days. In other words, the average was 209 labor days, and 344 days of residence for each person.

It is scarcely possible to present a more favorable bill of health. Whoever heard of a manufacturer of white lead remaining in its manufacture during 344 successive days? Besides, most of the above workmen still remain, and are able to count upward of 1000 labor days.

Dr. Bouchut has carefully studied what should be called the *effects* rather than the *diseases* caused by this species of manufacture. They are as follows:

1. Pains in the throat and slight cough occur during the first days of labor, until the mucous membrane becomes accustomed to the exhalations from the white zinc. But they disappear very soon, and the workmen there are no more subject to cough and throat affections than the same given number of any other persons.

2. Many of the workmen are at various periods subject to a curious species of innervation, shown by febrile or non-febrile restlessness at night. But this does not affect the general health, and they return in the morning to their labor. Occasionally, there is a species of excitement, temporary, such as Delaroche and Barbier ascribe to the oxide of zinc, but with most it is the short feverish feeling just described. It is always of short duration, never dangerous, and disappears after the system has become accustomed to the employment.

3. Occasionally eruptions appear on the skin, of reddish papulæ, which readily disappear with proper treatment.

Having thus noticed the effects, Dr. Bouchut proceeds to mention three cases of slight disease, ascribed to this cause. But a careful analysis proves that they were not owing to it.

Here, then, we have results which are frequently produced by emanations from the *most harmless substances*, when inhaled in the form of powder. The difficulty only extends thus far. But while white lead, as powder, causes its severe results also, we must recollect that it is equally noxious when manipulated in the humid form. From this, however, white zinc is totally free. It is only the powder of it that affects the workmen.

We should also remember the large doses that have for many years been administered of the white oxide as a medicine, without causing any accident. M. Orfila, the highest authority in toxicology, gave 20 grammes ( ) to small and feeble dogs, with only the result of gentle vomiting, and a subsequent perfect recovery. How very different are the consequences of administering white lead.

As to the economic value of white zinc: it can be manufactured for exactly the same price as white lead, and being much lighter, a larger quantity can be sold for the same sum of money. It cannot be adulterated. This, indeed, has been made a formidable objection to it. White lead is very commonly mixed with sulphate of barytes, and not unfrequently with chalk. White zinc can be used with equal facility as a paint. It does not dry as readily as white lead, but the difference in time is small. It has been objected that it does not set well as a paint, but this is altogether a mistake. Two coats cover wood *very nearly* as well as white lead, and there is this further advantage, the vapors of sulphuretted hydrogen do not affect it, while all the preparations of lead turn black from them.

M. Leclaire, an eminent house-painter, and others, have verified its use, on more than two thousand buildings, some of them public ones, to the satisfaction of the community.

The results, then, of suppressing the use of white lead by public authority will be—to save annually the lives of eighty workmen—to prevent 2000 cases of disease, some of them, indeed, incurable—and to enable active industry to continue its labors uninterrupted.—*Abridged from La Lancette Francaise (Gazette des Hopitaux) American Jour. Med. Sciences.*

